# Implementing street triage: a qualitative study of collaboration between police and mental health services

**DOI:** 10.1186/s12888-016-1026-z

**Published:** 2016-09-07

**Authors:** Kimberley Horspool, Sarah J. Drabble, Alicia O’Cathain

**Affiliations:** School of Health and Related Research (ScHARR), University of Sheffield, 30 Regent Street, Sheffield, S1 4DA UK

**Keywords:** Street triage, Mental health, Police, Crisis, Response, Collaboration

## Abstract

**Background:**

Street Triage is a collaborative service between mental health workers and police which aims to improve the emergency response to individuals experiencing crisis, but peer reviewed evidence of the effectiveness of these services is limited. We examined the design and potential impact of two services, along with factors that hindered and facilitated the implementation of the services.

**Methods:**

We conducted 14 semi-structured interviews with mental health and police stakeholders with experience of a Street Triage service in two locations of the UK. Framework analysis identified themes related to key aspects of the Street Triage service, perceived benefits of Street Triage, and ways in which the service could be developed in the future.

**Results:**

Stakeholders endorsed the Street Triage services which utilised different operating models. These models had several components including a joint response vehicle or a mental health worker in a police control room. Operating models were developed with consideration of the local geographical and population density. The ability to make referrals to the existing mental health service was perceived as key to the success of the service yet there was evidence to suggest Street Triage had the potential to increase pressure on already stretched mental health and police services. Identifying staff with skills and experience for Street Triage work was important, and their joint response resulted in shared decision making which was less risk averse for the police and regarded as in the interest of patient care by mental health professionals. Collaboration during Street Triage improved the understanding of roles and responsibilities in the ‘other’ agency and led to the development of local information sharing agreements. Views about the future direction of the service focused on expansion of Street Triage to address other shared priorities such as frequent users of police and mental health services, and a reduction in the police involvement in crisis response.

**Conclusion:**

The Street Triage service received strong support from stakeholders involved in it. Referral to existing health services is a key function of Street Triage, and its impact on referral behaviour requires rigorous evaluation. Street Triage may result in improvement to collaborative working but competing demands for resources within mental health and police services presented challenges for implementation.

**Electronic supplementary material:**

The online version of this article (doi:10.1186/s12888-016-1026-z) contains supplementary material, which is available to authorized users.

## Background

An effective response to mental health crisis requires collaboration from health services and the police to ensure the safety of community dwelling individuals and provide emergency access to mental health support [1, 2]. Street Triage is an example of a collaborative model of care between these services which is being piloted or has been adopted in some areas of the UK. New models of care may lead to improvements in mental health crisis response, which is seen as sub-standard compared to responses to physical health emergencies [1, 3]. Individuals who have experienced mental health crisis have also voiced their dissatisfaction; 86 % of service users, surveyed between 2011–2014, felt they did not receive the right response to resolve their mental health crisis [4]. This dissatisfaction with crisis services may be linked to the historically high and varied use of Section 136 (S136) of the Mental Health Act (1983) in some areas of the UK [5], whereby; “A police officer who finds a person in a place to which the public have access, who appears to be suffering from a mental health disorder and to be in immediate need of care or control, and if the constable thinks it necessary to do so in the interests of that person or for the protection of other persons, to remove that person to a place of safety”. The use of police custody as a place of safety, which has received unanimous condemnation due to the stigma, association of mental health crisis with criminality, and a lack of safety [6, 7], was also common practice in some areas due to limited availability in health based places of safety or concerns regarding risk of violence or intoxication.

In the UK the adoption of the Crisis Care Concordat has united the efforts of organisations involved in the crisis pathway to improve the collaborative response to crisis care. At a local level this typically includes the NHS (National Health Service) trust(s) responsible for delivering mental health services, acute care and emergency service providers such as the police and ambulance service. The initial progress of the Concordat has been encouraging, with the development of local action plans to address urgent and emergency access to crisis, as well as access to support before crisis, and quality of treatment during crisis and recovery. The use of police custody as a place of safety, which remains a high profile indicator of crisis management, and which the Crisis Care Concordat aims to eradicate, has also reduced from 7,881 in 2012/13 to 3,996 in 2014/15 [8]. However, the rationale for continued research into effective mental health and police collaboration for crisis care is strong; An unintended consequence of moving from institutionalised mental healthcare to community based models, which rely on continual risk assessment and management to ensure welfare, will ultimately require multi-disciplinary support to manage high risk [9]; data suggests that police based places of safety may still have been used in 35 % of cases and the true extent of police involvement in mental health incidents is likely to be underrepresented in data related to S136 and places of safety [10]. Leading police officers corroborate that mental health incidents will continue to represent a core function of police services in UK society [11], some feel this demand continues to be exacerbated by a chronic lack of funding for existing mental health services and health based places of safety.

There is limited evidence available to support the clinical and cost effectiveness of many new models of collaborative crisis care developed under the umbrella of the Crisis Care Concordat [3]. Street Triage, which initially attracted UK home office funding for pilots schemes, and other models of care involving the Police are included in this criticism. A recent review [3] identified only one non-peer reviewed, primarily descriptive study [12] and the national evaluation of Street Triage pilots schemes provided limited insight into effectiveness due to a large amount of missing data and study design [13]. However, there are examples of international research on mental health and police collaboration [14–19] and Crisis Intervention Team (CIT) programmes in the United States, where police officers receive specialist mental training to respond to incidents [20]. Studies of CIT suggest police officers demonstrate better self-efficacy in their skills to handle mental health incidents, improved knowledge, better perceptions and a favourable attitude to mental health incidents [21]. There is also greater verbal engagement and lower levels of arrest [22], more referrals to mental health services [23, 24], and officers may use less force in response to mental health scenarios [25].

Street Triage services in the UK adopt a different model from the police-led CIT programmes which can be considered to a*’police-based specialised police response’* i.e. the police respond to the incident and liaise with mental health services as a secondary action [26]. The typology of crisis response in Street Triage is perhaps dependent on model of Street Triage implemented, which itself may be influenced by variation in local geography, mental health needs of the population and resource availability [13]. However, the existing typologies identified by Deane et al.[26] may not present an ideal fit with current Street Triage models. For example; a *‘police-based specialised mental health response’* where a mental health worker is employed and embedded within the police organisation is similar to Street Triage services which utilise a mental health worker in a police control room to take telephone calls and/or share information with police officers, but the mental health worker is likely to be employed by the local NHS trust. Similarly, a *‘mental health- based specialised mental health response’* where a mobile crisis response based within mental health services but linked to the police and attends the scene of a crisis, again is a common model in Street Triage, yet response is likely to be based within the police and supported by the mental health service as the police have responsibility for the initial call handling and the dispatch of a vehicle to the scene.

Street Triage, in the broadest sense, aims to improve access to mental health services for individuals in contact with emergency services [13]. At a local level this may be achieved with a variety of core objectives which include; improved service user experience, access to the crisis pathway, improved working relationships with health and emergency services, as well as reductions in the use of police custody as a place of safety, reduced repeated use of S136 and reductions in the use of health based places of safety, conveyance and attendance at emergency departments, and the avoidable use of staff from mental health and emergency services in the crisis pathway [13]. The complexity and challenge to achieve these objectives in Street Triage requires further investigation as the evidence for a reduction in use of S136 is equivocal [27–29], yet qualitative investigation has demonstrated mental health and police services offer positive accounts of Street Triage [28–30].

This current study builds on the qualitative evidence on Street Triage by reporting stakeholder interviews with mental health services and the police from a Street Triage service in two locations in the UK. Specifically, we aim to explore the design and operation of Street Triage and identify potential barriers and facilitators affecting its implementation.

## Method

### Design

A cross sectional qualitative interview study was undertaken on a Street Triage service being piloted across two geographical locations in the UK. The Street Triage services were opportunistically identified for evaluation but considered to merit investigation due to their independence from UK government funded pilot schemes and their uniqueness in that the Street Triage service was linked by the same police service but operated a different Street Triage service across the two locations. The services were also piloted at different times: location 1 first piloted the service from November 2012-April 2013 and Street Triage was then adopted as standard practice, and in location 2 a single 10- week pilot was conducted between January 2014 and March 2014, the Street Triage service then ceased. The service in location 2 also only provided partial coverage to area. Interviewing was completed between September 2014 and January 2015.

### Participants

Senior representatives in the police and mental health services in two locations supported purposeful sampling [31] to identify stakeholders who met the study eligibility criteria: individuals with managerial or front-line operational experience of the Street Triage services. Police stakeholders were recruited from the single police force running the scheme, whereas mental health stakeholders were recruited from two NHS trusts. To preserve the anonymity of participants in the study the name of each location has been omitted and will be referred to as location 1 and location 2, characteristics of these locations is found in Table 1. Opportunistic promotion of the study to employees from mental health services and the police was also conducted at a Street Triage briefing (as described in Table 3) in location 1.

**Table 1:** Mental health and policing profile in Street Triage locations

	Location 1	Location 2^a^
Population	138,400	1,759,800
Location size (sq mi)	146.8	1,622
Estimated % of population 16–74 with severe mental illness (national comparison 1.1 %)^2^	0.8 %	0.6 %
% of people living in 20 % most deprived locations (national comparison 20.3 %)^c^	5.7 %	3.7 %
Number of S136 detentions by MH provider 2013/2014 ^b,c^	60	250
Number of S136 detentions by police force boundary in 2013/2014^c^	805	
% of all S136 detentions that result in individual being taken to a health based place of safety^c^	57.8 %	

^a^The Street Triage service did not operate across the whole location

^b^Recorded in the Mental Health Minimum Data Set (MHMDS)

^c^ http://www.cqc.org.uk/content/thematic-review-mental-health-crisis-care-initial-data-review [41]

The researcher (KH) approached potential participants via email which included a study invitation with an information sheet and consent form. Non-responders to the initial invitation received up to two reminder emails. Ethical approval for the project was obtained from the School of Health and Related Research Ethics Committee, University of Sheffield (Reference number: 0756). Permissions to undertake the study were obtained from the NHS trusts and the police force.

Twenty- six individuals were invited to participate in the study (police *n* = 13, mental health services location 1 *n* = 5, mental health services location 2 *n* = 8), 18/26 responded positively and a greater proportion of the non- responders were from mental health services (6/8) and from mental health services in location 1 (3/5). Interviews were conducted with 14 participants, either in person (*n* = 4) or over the telephone (*n* = 10). The average duration of the interviews was 45 min, ranging between 26 and 75 min. Four individuals who initially responded positively did not complete an interview due to the unavailability of the interviewee (*n* = 1), lack of written informed consent (*n* = 1), or were declined by the researcher as the interviewee characteristics were already well represented in the sample (*n* = 2). More interviewees were from the police (9/14), male (9/14), and could be described as working operationally on the front-line rather than having managerial responsibilities (8/14) (Table 2). A greater proportion of interviewees in location 1 were from the police (5/7) whereas location 2 had a more balanced representation from mental health services and police (3/6 respectively).

**Table 2:** Participant demographic data

Unique identifier	Gender	Organisation	Job title	Managerial role in street triage	Location
01	Male	Mental Health Services	Register Mental Health Nurse- Crisis team	No	1
02	Male	Police	Police Constable	No	1
03	Male	Mental Health Services	Inpatient Modern Matron	Yes	1
04	Male	Police	Police Constable	No	1
05	Male	Police	Police Sergeant	Yes	1
06	Female	Police	Police Constable	Yes	1
07	Male	Mental Health Services	AMHP Manager	Yes	2
08	Male	Police	MH inspector	Yes	1 & 2
09	Male	Police	Police Sergeant	Yes	2
10	Female	Police	Police Constable	No	2
11	Female	Mental Health Services	Community Psychiatric nurse	No	2
12	Male	Police	Police Constable	No	2
13	Female	Mental Health Services	Crisis worker	No	2
14	Female	Police	Police Constable	No	1

### Data collection

A semi-structured topic guide (see additional file 1) was developed and piloted to ensure data collected addressed the aims of the study and that the questions were understood. During interviews, emerging topics of interest, for example the discussion of wider changes within mental health and policing, were followed up to gain a deeper understanding of the context and considerations for implementing Street Triage [32]. It was not necessary to conduct any repeated interviews. Interviews were conducted confidentially, face-to-face at stakeholder workplaces or by telephone. The interviewer was a female health services researcher educated to MSc level with experience of undertaking semi-structured interviews and an unbiased interest in mental health crisis response (KH). Interviews were conducted until data saturation was achieved and the sample reflected the characteristics of the Street Triage services. Field notes taken during the interview where used to prompt discussions and audio recordings were transcribed verbatim and checked for accuracy by a separate researcher (SJD) prior to analysis.

### Analysis

A Framework analysis was conducted to identify themes related to the implementation of Street Triage service using Nvivo qualitative software (v.10, QSR International 2014). This approach allowed for identification of a priori and emergent themes [33] and is utilised in applied policy research as well as mental health studies [34]. The approach also supported researchers to systematically consider the similarities and differences within and between cases which was important considering the separate organisations and locations represented in the sample [35]. Two researchers (KH and SJD) participated in the process of familiarisation by reading transcripts and undertaking regular discussion to identify a thematic framework of issues specific to the research aims (e.g. service configuration) and themes identified inductively from the transcripts (e.g. impact of the service). These themes were then expanded to create a framework of key themes and linked sub-themes (KH). Further discussion between researchers (KH, SD, AOC) resulted in revision to the framework and KH conducted the indexing where revised themes and emerging minor subthemes were applied to all transcripts. Matrices of themes, wherein stakeholder characteristics were classified according to gender (male/female), location (1 or 2), organisation (mental health services or police) and role (operational or managerial) facilitated the process of data synthesis into the key messages (KH).

## Results

### Overview of themes

Three themes were identified: (1) the key aspects of a Street Triage service (2) the perceived benefits of Street Triage, and (3) ways in which the service could be developed in the future. Within these themes, considerations for Street Triage design, and facilitators and barriers to the implementation of services were identified.

### Key aspects of a street triage service

#### Street triage operating models

The development of pilot services was led by the police in both locations but was supported by both police and mental health managers. Once the Street Triage team was operationalised, a ‘bottom up’ approach to service development was adopted using feedback from front-line staff to influence design and re-evaluate objectives. The resulting operating models, which we use as an overarching term to describe characteristics of service design (e.g. operating hours) and the individual service components (Table 3) differed by location. Although both locations involved a mental health worker responding to incidents in police cars, and held briefing sessions for relevant stakeholders (police, mental health and social care colleagues) to inform them about how the service worked, they differed in two ways. One location also had a mental health worker in the police control room and the other had a recovery programme for frequent service users. Services were also implemented at different times during the week, based on perceived demand. For example in location 1 the Joint Response car was in operation on Wednesday, Friday and Saturday from 17:00–01:00 whereas in location 2 the control room was in operation on Thursday, Friday and Saturday from 18:00–02:00.

**Table 3:** Components of the Street Triage services

Components of the service	Description	Location 1	Location 2
Joint Response Car	Mental health worker and police officer visit to the individual in MH crisis by travelling in the same vehicle	x	x
Mental health presence in police Control Room	Mental health worker handles incoming calls from individuals who need mental health support, as well as providing advice to police colleagues		x
Integrated Recovery Programme	Multi-agency intervention for service users who create a high demand on police and mental health services	x	
Street Triage Briefing sessions	Educational sessions for police and mental health staff directly involved in the implementation and management of the service, as well as staff from wider organisations which work alongside police and mental health services e.g. social care	x	x

#### Tailored service for local need

Interviewees emphasised there was no ‘one size fits all’ for service design and the operating model should take into account the geography and population size of a location. For example, interviewees felt that location 1 was better suited to the Joint Response Car because this was a small geographical location where rapid attendance at the scene was achievable, whereas in location 2 the presence of mental health support in the control room covered a larger county and was supported by an additional Joint Response Car in the most densely populated city:

*“It’s too big a location and you’d take half an hour to get from one to the other. And that’s just not reasonable or realistic. So if I had a magic wand and an unlimited budget, I would get the worker back in the control room. I’d get the car back on the streets of [City] and I would get one in the east. But you know with all the will in the world it would not be worth putting one in the north. It just wouldn’t be worth it”* (08, police, manager, location 2)

Despite consideration of the geographical factors in the design of the service, the environment did present some barriers such as limited mobile phone coverage to communicate with colleagues (location 1), and high volumes of traffic (location 1). Some stakeholders also had no previous experience of working together as they were routinely based in different towns (location 2), and felt this impacted the quality of the Street Triage response and also posed challenges for effective collaboration outside of Street Triage hours:

*“I always find it difficult to get in touch with my own service to get that information… we have got a little mobile phone but because of the location…there are dead spots you know and the other thing is we are always very busy and when I am phoning I get engaged… so I am out there with a patient with the police trying to sort things out so it’s very difficult you know to get my own service sometimes”* (03, mental health worker, manager, location 1)

#### Referrals to mental health services

Outside of Street Triage, police and mental health service interaction was complex in location 2 because police service boundaries encompassed many NHS trusts and police officers would need to identify which NHS Trust was responsible for care when responding to a mental health incident:

*“Actually in the north of [Location 2] we are covered by [NHS Trust #1]… It gets very complex up there but in the south you know you have got [NHS trust #2], [NHS Trust #3], you have got [NHS trust #4]. You have got almost… I don’t know actually how many there are but you have got a lot”* (09, police, manager, location 2)

During Street Triage hours a core function for mental health staff was to refer people to existing mental health services which required mental health workers to have knowledge and access to the appropriate services e.g. community based mental health teams. This was more easily facilitated if the mental health workers were familiar with services because they had worked with them previously:

*“I can refer them back to their care co-ordinator, I can refer them back to their GPs for possible referral to mental health services. Or it may be the fact that they are just feeling depressed and they need some antidepressants so refer them back to their GP, and I can refer them onto < Place > which is our community mental health service”* (03, mental health worker, manager, location 1)

***“****I worked within that team and understood the process, it was obviously probably much easier for us. So I would be able to write up my notes and the plan and then put, add on to the schedule for the next day that person to be called or contacted”* (13, mental health worker, front-line, location 2)

However, referrals did not mean that service users received the mental healthcare or treatment. Some mental health services had long waiting times and there was a limit to what the mental health workers could guarantee the patient would receive. Referrals also had the potential to cause an increased workload for other services:

*“It was always at the very least it would be telephone contact. That would be the only bit that I would guarantee because I couldn’t always guarantee a visit. I could always say to my colleagues I think that would be appropriate and that’s needed and put my rationale but that would be at the discretion of the team the next day to kind of look at their workload and how manageable it was”* (13, mental health worker, front-line, location 2)

#### Appropriate staff and rostering

Stakeholders described police and mental health workers on Street Triage as compassionate and motivated to work on the service; these attributes were seen as important to the success of the service. Some had been handpicked by managers, whereas others had volunteered to take on Street Triage duties. Having the appropriate knowledge and experience to handle mental health crisis was also seen as important for individuals from mental health services:

*“You need to have it that people want to do it, I don’t think it should be rota’d [rostered for all staff]…as I said before you know it’s better to have people want to do it than are forced to do it or told they have got to do it, so I think that’s the other thing that other locations need to look at… I think the other thing is that you need to have people, mental health practitioners who have quite a good knowledge of mental health issues, got some experience behind them as well. I don’t think that somebody who has just come out of finishing their training would in my opinion have that knowledge to go out”* (03, mental health worker, manager, location 1)

The demand for appropriate staff for the Street Triage service could cause problems for existing mental health teams because the Street Triage staff were sourced from existing mental health teams. Work on Street Triage meant that there was less resource available to deliver other aspects of mental health services:

*“…three practitioners were taken from our normal team so we weren’t extra resources. So we were three full time practitioners that would normally do nights and weekends. That pulled us out of an already stretched team. So I think that [project] was given a lot of priority …but I know that it put huge strain on our colleagues and at times, because we would have been on the night shift”* (13, mental health worker, front- line location 2)

The police approach to staffing the Street Triage service was adapted during the piloting and routine adoption of the service in location 1. Initially during the pilot period officers were paid overtime for additional Street Triage duties but when the service was adopted as a ‘routine’ Street Triage was staffed by an officer from the regular shift. This was also the approach adopted in location 2. Some interviewees felt at busy times losing one officer from the shift compromised their ability to respond to incoming police incidents:

*“I think because we have had our shift numbers cut, officers on shift, I think there needs to be some sort of provision put in place because we are just so short. You know it’s a job for an officer to get time off on leave, let alone you know then we have to support [project name] as well”* (14, police, front-line, location 1)

Managers were aware of this criticism but justified the decision with statistics which supported that one officer dedicated to mental health incidents would reduce the workload associated with mental health for other colleagues on the shift.

### Perceived benefits of street triage

#### Helping people with mental health problems

The stakeholders expressed an overwhelmingly positive perception of the Street Triage pilots for helping people with their mental health issues/crises irrespective of their organisation, location or job role:

*“I’ve seen it work, and I’ve seen it help people”*

(12, mental health worker, front-line, location 2)

*“…projects like this have got so much potential value in them”*

(01, mental health worker, front-line, location 1)

*“It was a very worthwhile piece of work”* (06, police, manager, location 1)

However, some stakeholders felt this positive perspective was not shared by a minority of police colleagues, who felt that mental health should be the responsibility of the health service. Thus a less enthusiastic response from some officers may pose challenges to the implementation of services:

“*I don’t want to put them all into one category I mean there’s a mixed feeling of officers on the ground. I can’t be as optimistic about all of their points of view and their opinions. Some of them are very much, I’m a police officer, this is mental health. But just because there is a cultural way of thinking, doesn’t mean it’s the right way of thinking. And I’d say for as many people as think it’s not part of their job, there is probably a lot more that think actually if this is going to help me do my job better…then I’ll give it a go”* (06, police officer, manager, location 1)

#### Shared decision making

Stakeholders perceived that without Street Triage services, police officers often made risk-averse decisions about people in mental health crisis. This was seen to be influenced by a lack of training or knowledge about mental health, and also a perceived lack of organisational support should an individual self- harm or attempt suicide after contact with police.

*I think the police in general have a habit of being reasonably risk averse. And have a habit of perhaps not taking decisions. It’s maybe not been the police entirely but I think in any large organisation if you don’t take a decision you can’t be criticised for the decision you have taken* (12, police, front-line, location 2)

Risk-averse decisions by the police were thought to lead to an increased demand for inpatient mental health services, which could be inappropriate for patients, and use up valuable health service resources:

*“So there’s a huge number of people who are coming into hospital who don’t need to be there…that’s got a huge impact on an individual and actually if they don’t need to be part of that service… From a health management point of view, I’d say that- you know generally the Health service is fairly slim resources and our resources were being called to be used in a way that wasn’t necessary”* (01, mental health worker, front-line, location 1)

This was contrasted with a shared decision making approach in Street Triage, whereby joint attendance at an incident or the availability of mental health support via telephone moved responsibility for decision making away from the police and enabled police to utilise different options where they may have previously exercised S136 powers.

#### Improving understanding between organisations

Stakeholders perceived that collaborative working at operational and managerial levels in the Street Triage resulted in improved understanding between the different organisations by dispelling misconceptions and building rapport between operational staff:

“*It just allowed us to understand what each role involved and how we could help that person. It was interesting in that respect and also if things did go a bit wrong then we were there to support the mental health workers if they needed it. So it’s definitely helpful in that respect, and understanding each other’s roles, it’s like oh right I didn’t realise you did that. So it was a huge learning curve”* (10, police, front-line, location 2)

*“I did go to the police station because I think it was really useful in terms of networking… breaking down some barriers because I think, you know, the police historically perhaps some attitudes towards mental health, there were perhaps some misunderstandings”* (13, mental health worker, front-line, location 2)

#### Improving information sharing policies

Managerial staff highlighted how establishing Street Triage services had initiated a review of local agreements related to collaborative working with mental health, police and emergency care and where necessary these were improved. For example, a new information sharing agreement clarified the type of information that could be shared, and enhanced access to information which was felt to improve police decision making both during and outside of Street Triage hours:

*`"Information sharing is the key, before [project name] I could have never have phoned them up and said I’ve this person here can I have some information to let me make a decision about what to do, I couldn’t have done that. But now I can make a phone call and say hi its < NAME > can I talk to you about this person and they can give me some information… before you know I have limited information I might have had to 136 them but now… they might give me more information to make an informed decision”* (04, police, front-line, location 1)

*“I think it was something that we very early on thought about, the information sharing agreement… I would often get complaints that the police had rung, they’re at a situation, and they’d rung to try get some information about somebody…And after that, you know, I don’t hear that, I think that’s had knock-on effects outside of [project name], that people can potentially more freely talk to each other without fear of being called to book about being confidential”* (01, mental health worker, front-line, location 1)

However, despite new information sharing agreements, there were examples of incompatible technology hampering the expansion of the service above and beyond trust and geographical boundaries:

*“They use different IT, completely different IT. At the moment they use two different versions of [computer system] but [city 2] have now moved away from [computer system] altogether. So it will be very, very difficult, even though the AMHPS [Approved Mental Health Practitioners] will regularly cross borders… That’s not the issue and the trust boundary isn’t the issue, it’s the IT”* (08, police, manager, location 2)

### Service development and future directions

#### Focus on frequent service users

Location 1 developed an ‘Integrated Recovery Programme’ to address the high demand created by frequent service users who would regularly call the police and mental health services for support:

*“…we’ve seen, if you have a good partnership that focuses on the street triage, you then automatically start talking about well actually we could do with a nurse there, or a mental health professional there….. So street triage is a vital first response tool, but without those mental health pathways, those critical points in the pathway, you are just going to have response, because you will be scooping up the same people. And it will be the same people coming round into the street triage again”* (05, police, manager, location 1)

This additional mental health intervention was described by police managerial stakeholders as reducing numbers of calls to mental health and police services thus providing potential long-term cost savings. However, the rapid development of the Integrated Recovery Programme under the umbrella of Street Triage created some concern from front-line staff from both organisations in location 1, as it was felt this would shift the focus of attention and resources away from the joint response vehicle which was held in high regard but had faced existing challenges to ensure the vehicle was manned during operational hours.

#### Reducing police involvement in mental health crises

A long-term aim described by a manager within the police was to change the landscape of responsibility in crisis response, to a situation where a police response is only required in the presence of immediate safety concerns for a client or public::

*“So one of the objectives of [project name], is the long term, almost a generational piece of work, to actually place mental health risk, mental health responsibility, back into the NHS, because it has never been in the NHS, because culturally the wider thing is the police in the UK scoop up everything.”* (05, police, manager, location 1)

The rationale given was that this would result in better patient care, that the police lacked skills to deal with this client group, and that it would reduce the high workload burden on the police. These were also identified as the initial drivers for developing Street Triage by both police and mental health interviewees. The police interviewees understood that moving the workload to health-based services would be challenging for technological reasons, such as incompatible computer systems and limitations in the call transfer systems, but the key challenge would be the absence of additional funding for mental health services to perform this function.

## Discussion

We present some of the earliest qualitative findings of the feasibility and stakeholder acceptance of Street Triage in the UK, and demonstrate that stakeholders from mental health services and police strongly support Street Triage collaboration and perceive it to benefit individuals suffering mental health crises in the community. Street Triage services included a variety of components to address the initial response to mental health crisis, and developed further to include interventions targeted at service users who contact mental health and police services more frequently. The design of services was shaped by the geography of the local location, with consideration of the challenges posed by local location size and population density. A key function of the Street Triage service was to refer service users to existing mental health services. Mental health staff with pre-requisite experience and knowledge of crisis response working in Street Triage was perceived as key to its success but appropriate staffing arrangements presented challenges for mental health and police managers. Perceived benefits of Street Triage included a reduction in risk averse decision making by police as a result of greater shared decision making in Street Triage, a greater understanding of stakeholder roles in crisis response and also the development or improvement of local agreements to facilitate collaborative working, such as an information sharing agreement. Future directions of Street Triage identified by police included a focus on reducing frequent callers and a longer term desire to reduce police input for mental health incidents where there are no immediate safety concerns.

The design of the Street Triage services in the current study is consistent with Street Triage design in other localities where a joint response in a dedicated shared vehicle and mental health practitioner support in the control room have been piloted together [27, 28] or separately [12]. This also identifies that a number of different collaborative operational models may be labelled as ‘Street Triage’ [10], but could also be argued as standalone collaborative interventions for example mental health training for police offers. Our findings also support those of Irvine, Allen and Webber [29] in that Street Triage services in the UK demonstrate some crossover between crisis response typologies [26], which may suggest that further assessment of existing Street Triage operating models should be taken to develop new terminology applicable to UK services. Previous studies also corroborate our stakeholders’ perception that the joint response vehicle was more effective when covering a smaller area with a dense population, compared to the greater reach of mental health support via a police control room [28, 30] and the complexities of cross boundary working [36].

An important function of Street Triage was to refer the service user to a health based service most appropriate for their needs. This has been identified widely as a key mechanism for collaborative crisis response models [27, 29, 30, 37–39]. In the current study mental health stakeholders discussed making referrals to community mental health services which is a pattern supported by Oxfordshire, UK, Street Triage referral data [28]. However Heslin et al. [27] identified the majority of referrals in a different service were to General Practitioners and emergency departments. This is interesting to consider further because Street Triage service users are frequently known to mental health services prior to the Street Triage encounter [30], and also because the specialised, immediate mental health care required by an individual in crisis may be unobtainable via general practitioners, thus impacting clinical and cost effectiveness and service user satisfaction. Alternatively, if Street Triage service users are not deemed to require immediate crisis support the core objectives of Street Triage may require review or ‘gate-keeping’ for service access may be considered.

The examination of barriers and facilitators to Street Triage in the current study raised two somewhat contradictory ideas on the short and long term impact of Street Triage which Street Triage leaders, commissioners and researchers may wish to consider. This related to the potential of 1) short term competing demands, due to increased need for staff and care from existing services and 2) long term improvements to collaborative working practices. Firstly, stakeholders interviewed in this study perceived that the implementation of Street Triage increased the demand on mental health services and police to allocate appropriate staff for Street Triage duties which may have impacted staffing and capacity of existing services. The idea of competing demands is not unique [28, 36] and is compounded by challenging financial situations for both health services and the police. For the mental health service this issue could also be compounded by the additional demand created by Street Triage referrals. This additional demand for appropriate staff, who are key to the facilitation of Street Triage [16, 29], represents a serious consideration to ensure that pressures in mental health crisis response are not moved to other services in the crisis care pathway. Secondly, and more positively, we present the potential for Street Triage to produce long term benefits to collaborative working between mental health services and police. This related to reduced risk-averse decision making by police as a result of shared decision making [28, 29, 37, 40], improved understanding of roles and responsibilities between organisations and improved local agreements such as information sharing policies [29]. Interestingly, these changes may not be dependent on the limited availability and reach of a joint response vehicle, and thus have the potential to impact collaboration and improve patient care outside of core Street Triage hours and on other shared priorities such as frequent service users [30].

### Limitations

The small sample of stakeholders included in the study had limitations. We did not include the perspectives of service users who have received the Street Triage service, or their family and carers. The sample also did not include the perspectives of wider healthcare services such as the ambulance service, emergency departments, or secondary services; although they were not active partners in the Street Triage service they may have been affected by the service. Finally, although it could be argued the sample had lower representation from mental health services than police we felt this reflected the police leadership in the development of the service. Senior stakeholders supporting our sampling may also have resulted in bias interviewees who had positive predispositions to the service. Our reflections on this lead us to believe that this was unlikely because stakeholders appeared to be candid in their responses during interviews and highlighted challenges for Street Triage as much as positive views.

## Conclusions

We add to evidence which demonstrates support for Street Triage, the design of which was perceived to be informed by the local geography and population density. Stakeholders perceived Street Triage to be helpful for individuals experiencing mental health crisis by enabling service users to be referred to mental health services which offered an alternative to the police use of Section 136. Skilled mental health staff with experience of crisis response, and motivated individuals from both organisations was perceived as facilitating the implementation of the service. We suggest there is potential for Street Triage to facilitate long term improvements to collaborative working, by allowing greater understanding of roles, shared decision making, and local information sharing policies. However, the development of services with limited resources may present a challenge to managers, particularly within the mental health services, due to the increased demands for appropriate staff and potential for increased workload to community mental health teams. Future research should examine whether Street Triage service users consider it to be an acceptable and effective response to their mental health crisis. Robust evaluations to determine effectiveness should also consider the impact of variation in referral behaviour as well as the impact of Street Triage on the whole crisis system.

## Additional file

Additional file 1: ‘Interview topic guide’. Questions and prompts used to obtain information on the Street Triage service. (DOCX 17 kb)

## Abbreviations

AMPH: Approved Mental Health Practitioner
NHS: National Health Service
S136: Section 136 of the Mental Health Act 1983
