# Supplementary material for: Implementing street triage: a qualitative study of collaboration between police and mental health services
Source: BMC Psychiatry. 2016 Sep 7;16(1):313. doi: 10.1186/s12888-016-1026-z (PMC5015199; doi:10.1186/s12888-016-1026-z)
Supplement: Additional file 1: — ‘Interview topic guide’. Questions and prompts used to obtain information on the Street Triage service. (DOCX 17 kb) [file 12888_2016_1026_MOESM1_ESM.docx]

**Street Triage Interview Topic guide**

1. Could you describe the Street triage service, and where your role fits into the service?
2. When the Street Triage service is available, what is different about how a situation is dealt with? Or how you respond?
3. What do you think are the aims of the Street Triage service?
4. Do you think the Street Triage service is a useful way of working?

- In comparison to other initiatives?

e.g. information sharing, better training, telephone triage, liaison and diversion,

1. What helps the service to run well?
2. Are there any difficulties working in this way?
3. What would indicate to you that the Street Triage service is successful?

-For police/MH services

- for service users/patients

8. The initial data available on the service suggests there has been a decrease in the use of S136 powers, what do you think about this?

9. If you could offer any advice to other areas looking to adopt Street Triage, what would that be?

10. Is there anything which we haven’t mentioned about Street Triage which you think is important to discuss?
